# Self-Assembling Peptide Nanofiber Scaffolds Accelerate Wound Healing

**DOI:** 10.1371/journal.pone.0001410

**Published:** 2008-01-09

**Authors:** Aurore Schneider, Jonathan A. Garlick, Christophe Egles

**Affiliations:** 1 Division of Cancer Biology and Tissue Engineering, Department of Oral and Maxillofacial Pathology, Tufts University, School of Dental Medicine, Boston, Massachusetts, United States of America; 2 Department of Biomedical Engineering, Tufts University, Medford, Massachusetts, United States of America; 3 Center for Biomedical Engineering, Massachusetts Institute of Technology, Cambridge, Massachusetts, United States of America; Massachusetts Institute of Technology, United States of America

## Abstract

Cutaneous wound repair regenerates skin integrity, but a chronic failure to heal results in compromised tissue function and increased morbidity. To address this, we have used an integrated approach, using nanobiotechnology to augment the rate of wound reepithelialization by combining self-assembling peptide (SAP) nanofiber scaffold and Epidermal Growth Factor (EGF). This SAP bioscaffold was tested in a bioengineered Human Skin Equivalent (HSE) tissue model that enabled wound reepithelialization to be monitored in a tissue that recapitulates molecular and cellular mechanisms of repair known to occur in human skin. We found that SAP underwent molecular self-assembly to form unique 3D structures that stably covered the surface of the wound, suggesting that this scaffold may serve as a viable wound dressing. We measured the rates of release of EGF from the SAP scaffold and determined that EGF was only released when the scaffold was in direct contact with the HSE. By measuring the length of the epithelial tongue during wound reepithelialization, we found that SAP scaffolds containing EGF accelerated the rate of wound coverage by 5 fold when compared to controls without scaffolds and by 3.5 fold when compared to the scaffold without EGF. In conclusion, our experiments demonstrated that biomaterials composed of a biofunctionalized peptidic scaffold have many properties that are well-suited for the treatment of cutaneous wounds including wound coverage, functionalization with bioactive molecules, localized growth factor release and activation of wound repair.

## Introduction

Skin functions to provide a physical and chemical interface that protects the host against invasion by toxins and microorganisms and prevents dehydration that can result from loss of barrier function. The loss of skin integrity and function due to wound injury has led to efforts designed to better comprehend the molecular and cellular mechanisms that can optimize wound repair [1], [2], [3]. The complex nature of wound healing requires the migration and proliferation of keratinocytes that are temporally-regulated by numerous growth factors and their receptors that are upregulated in the wound environment [4], [5], [6]. The complexity of the wound environment has been recreated in human, bioengineered *in vitro* 3D tissues known as human skin equivalents (HSE), that have many morphologic and phenotypic properties of human skin. Adaptating HSEs to study wound reepithelization has demonstrated several key responses including cell proliferation, migration, differentiation, growth factor responsiveness and protein expression that mimic the response to wounding seen in human skin [7]. In light of this, we used HSEs to study the effect of a new growth factor-releasing biomaterial on wound reepithelialization.

As a mediator of wound repair, Epidermal Growth Factor (EGF), is involved in epidermal regeneration by stimulating the proliferation and migration of keratinocytes at the wound edge [8], [9], [10], through its interaction with high affinity receptors on both fibroblasts and keratinocytes [11], [12]. EGF is thought to play a critical role in wound repair during the first few days after injury, until wound reepithelialization is complete. Due to its relatively short half-life of one hour [13] and its turnover results in loss of occupied receptors and decreased activity [12], it is necessary to apply EGF frequently to a wound to maintain an effective local concentration during initiation of wound healing [13]. Therefore, topical EGF application that would result in the sustained flux of EGF into the wound environment would be a very effective way to locally deliver EGF. To achieve this therapeutic goal, it would be optimal to deliver biologically-meaningful doses of EGF in a wound dressing. Such a bioactive dressing should fulfill several criteria for its optimal function: 1) biocompatibility in the absence of cytotoxicity, 2) easily applied to the wound, able to conform to the wound surface, and easily removable after healing, 3) provides a moist environment and protects the wound against dehydration, 4) allows gas exchange between the wounded tissue and the external environment, 5) biofunctionalized to allow the slow release of bioactive agents.

In consideration of these criteria, we have studied if a novel, self-assembling peptide (SAP) nanofiber scaffold combined with EGF, could serve as a bio-active wound dressing. SAP nanofibers have already been shown to serve as excellent materials for a variety of controlled, molecular-release applications [14], [15]. The individual nanofiber consists of ionic, self-complementary peptides with 16 amino-acids (RADA16-I, Ac-RADARADARADARADA-CONH2) that undergo self-assembly into hydrogels containing 99.5% w/v water when exposed to physiological media or salt solution. These scaffolds closely mimic the structure and porosity of extracellular matrices in that growth factors and nutrients freely diffuse in and out of the scaffold at very slow rates [15]. A wound dressing comprised of SAP scaffolds could be optimized by directly molding it to the wound surface as a hydrogel.

In the present report, we have used wounded HSEs to study the capacity of SAP scaffolds that are combined with EGF, to modulate the wound healing rate in tissues that closely mimic the human wound response *in vivo*. We found that EGF is released from the hydrogel only when it is in contact with the wound. Released EGF increased the rate of wound closure by more than 3.5 fold when compared to wound closure under an SAP scaffold without EGF. Moreover, the increase in reepithelialization due to SAP-EGF was mostly due to cell proliferation as demonstrated by BrdU incorporation. These results demonstrate that this new bioactive material has many properties that would allow it to be effectively applied directly to a wound to accelerate reepithelialization of non-healing, chronic wounds and has significant potential as a wound repair agent.

## Results

### Design of Self-assembling peptides to incorporate EGF

**Figure 1:**
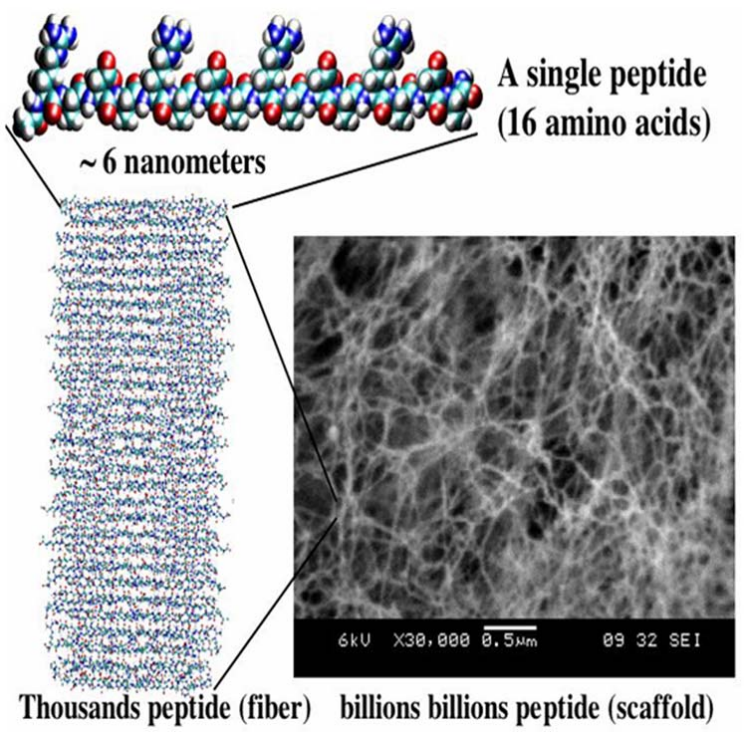
A designer self-assembling peptide nanofiber scaffold. A single peptide, approximately 6 nanometers, is shown. Thousands of peptides self-assemble to form a single nanofiber, trillions of peptides or billions of nanofibers form the scaffold that contains ≈99.5% water and 0.5% peptide materials [16]. Positive and negative charges are labeled in blue and in red, respectively.

Figure 1 depicts the sequence of the SAP RADA16-I that has been used for this study. The self-assembling peptide scaffold is composed of short, 8- to 16-residue (≈2.5–5 nm in length) peptides which are chemically synthesized and form extremely stable β-sheet structures in water [16]. The scanning electron microscopy image shows the scaffold after the assembly of all of the polypeptides (Figure 1). These peptides not only self-assemble to form stable nanofibers, but also form higher-order nanofiber scaffolds, namely hydrogels with high water content (>99.5 (wt/vol)% water). The gelation process is charge dependent, accelerated either by changing to a neutral pH or by adding physiological concentration of salt solutions.

### Preparation of the HSE for the Wound healing model

**Figure 2:**
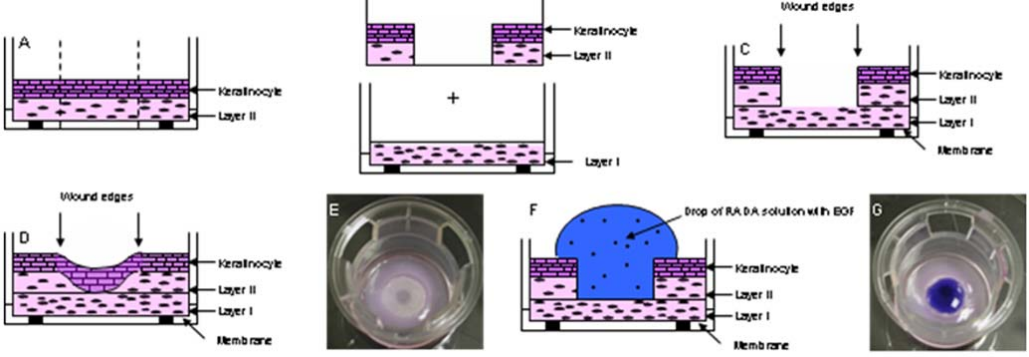
Construction of composite organotypic co-culture wound healing model. A) Schematic drawing of stratified keratinocyte sheet growing on contracted collagen matrix containing fibroblasts (Layer II). This skin equivalent culture rests on a semi-permeable membrane and is nourished with medium from below, thus being exposed to an air-liquid interface. A wound is formed by punching a hole through the epithelium and collagen matrix. B) the wounded culture is transferred onto a second collagen matrix that has undergone contraction (Layer I). C) the resultant composite co-culture consists of two layers of contracted matrix and one layer of epithelium. Wound edges are seen at the transition zone from layer I to layer II and are noted with arrows. D) reepithelialization occurs as wounded keratinocytes migrate onto the collagen in layer II and is then followed by stratification of the tissue to reconstitute a fully stratified epithelium that covers the wound bed. E is a picture of the wound as depicted in C. on top of the wound a drop of self-assembling peptide containing or not the protein is added (scheme F). G is a picture of the wound covered by the drop of peptide stained with comassie blue.

Immediately after establishing a full-thickness, incisional wound in HSEs, epithelium at the wound edges underwent a sequence of coordinated temporal and spatial events that resulted in wound reepithelialization. Figure 2 presents a schematic diagram depicting construction of HSEs that have been adapted to study the response of wounded keratinocytes and to analyze the tissue phenotype during reepithelialization. We found that SAP nanoscaffolds could be formed directly on wounded or non-wounded control HSEs by adding a drop of peptide solution directly onto the upper surface of these tissues. Scaffolds rapidly formed a gel in less than 30 min and completely covered the wound surface (Figure 2, F and G) by filling the wound incision.

### Release of EGF from the SAP gels

**Figure 3:**
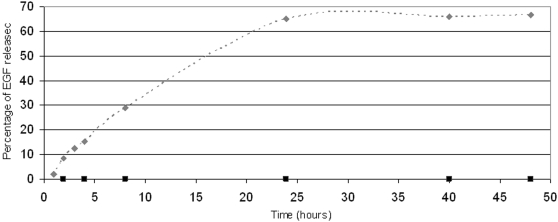
Percentage of EGF released from the RADA16-I gels, (⧫) when the gel is in contact with the wound, and when the gel is put in PBS (▪).

Before testing the effect of SAP-EGF in a wound, we first studied the rate of EGF release into the media supernatant when sampled at 1, 2, 3, 4, 8, 24, 40, 48 hours. As shown in Figure 3, no released EGF was detected in the supernatant by ELISA, demonstrating that the protein remained in the scaffold and was not released when the SAP was incubated in PBS.

To determine if EGF release occurred in the wound environment, SAP scaffolds were applied to the surface of HSEs after wounding and supernatants were collected at these same times. Under these conditions, EGF was detected in the media supernatant by ELISA and the percentage of EGF released from the RADA16-I gel over a 48 hour period was calculated (Figure 3). The EGF release profile was biphasic as there was a large initial release of EGF that was followed by a phase in which EGF was not released. The amount of EGF released (6.55 µg) reached a plateau by 24 hours and totaled 65.5% of the EGF that was present in the SAP. Our observations that EGF was only released when RADA16-I scaffolds were in contact with the wounded tissue, highlights the importance of intimate tissue contact with the wound environment in the release process.

### Accelerated Wound Reepithelialization by EGF Released from SAP

**Figure 4:**
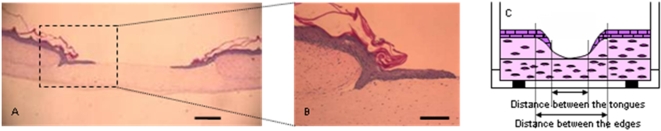
Hematoxylin and Eosin staining of wounds (A,B) shows epithelial tongues migrating across the wound bed to close the wound. C is a schematic figure of the wound. The distance between the wound edges and between the epithelial tongues is measured to determine the percentage of wound closure. The scale bar in A,B is 500 µm, on figure C the scale bar is 100 µm.

**Figure 5:**
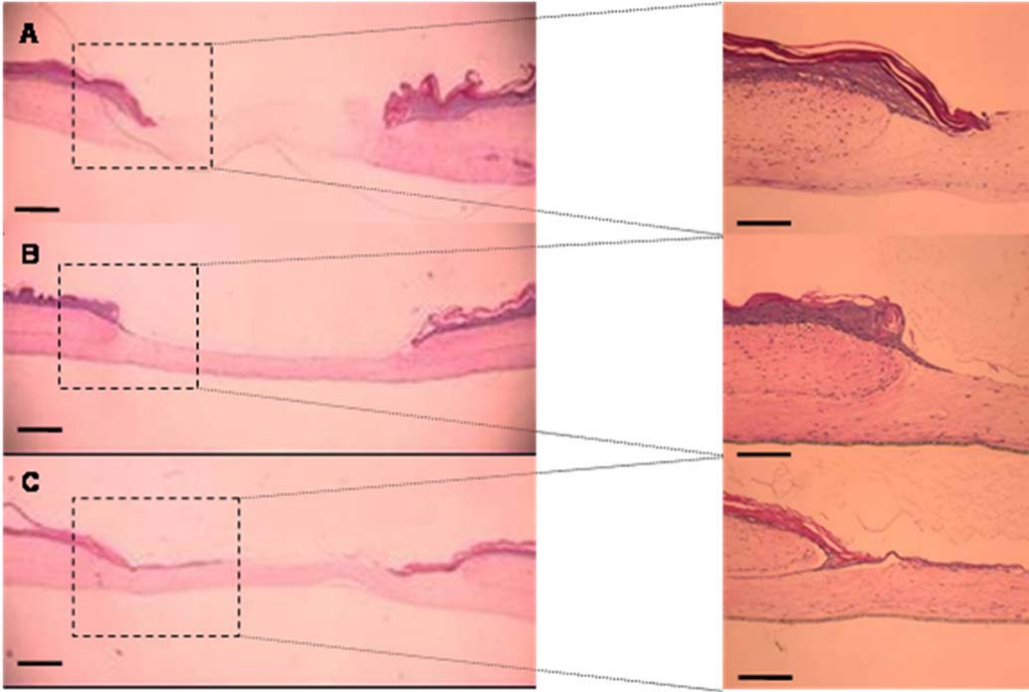
Hematoxylin and Eosin staining of the wound after 48 hours (A,B,C). Control tissues where nothing is added on top of the wound (A), are compared to wounds covered with a drop of RADA16-I (B and with a drop of peptide solution containing the growth factor EGF (C). The scale bar is 500 µm for wounds. For the inserts the scale bar is 100 µm.

**Figure 6:**
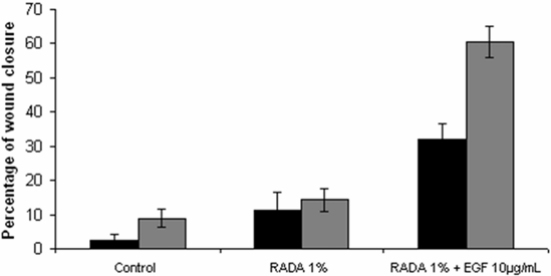
Percentage of wound closure after 24 hours (black bars) and 48 hours (grey bars). Data points are average of N = 7 and error bars represent±SD.

Tissues were harvested 24 and 48 hours after wounding and stained using hematoxylin and eosin for morphological analysis. Figure 4 C demonstrates the means by which wound closure was evaluated by measuring the distance between the wound edge and the tip of the epithelializing tongue on each side to assess the percentage of wound closure. To evaluate the effect of EGF on wound re-epithelialization, we compared wound closure of control tissues in which no RADA was added to the wound surface (Figure 5A) to either 1% RADA without EGF (Figure 5B) or RADA 1%+EGF 100 µL (Figure 5C). After 48 hours, both the control wound and the wound covered with the SAP scaffold had a similar response characterized by a short epithelial tongue that initiated migration at the wound edge. However, a significant increase in the rate of reepithelization was observed when control wounds without EGF were compared to wounded tissues covered with RADA16-I containing EGF. In order to quantify wound closure, we calculated the percentage of reepithelialization after 24 hours (black boxes) and 48 hours (grey boxes) (Figure 6). We showed that wounds covered with the SAP scaffold containing EGF had undergone a 3-fold greater degree of reepithelization than either the control or RADA16-I wounds. 48 hours after wounding this difference was even greater as wounds treated with EGF showed 60±4% closure, whereas control and the RADA16-I only exhibited wound closure of 9±3% and 14±3%, respectively. Our observation that SAP hydrogel alone increases wound healing rates could be explained by the fact that the presence of the SAP offers a moist and protected environment that supports early cell migration and wound closure. We also tested a positive control by adding EGF (100 µg/ml) directly to the culture medium. In response to this type of EGF delivery, the wound was closed by 57±5%, thus showing no significant differences with the SAP containing EGF. Thus, the presence of EGF incorporated into the SAP scaffold and released when in contact with the wound had accelerated wound closure as efficiently as EGF delivered in the media.

### Increased reepithelization linked to SAP-EGF

**Figure 7:**
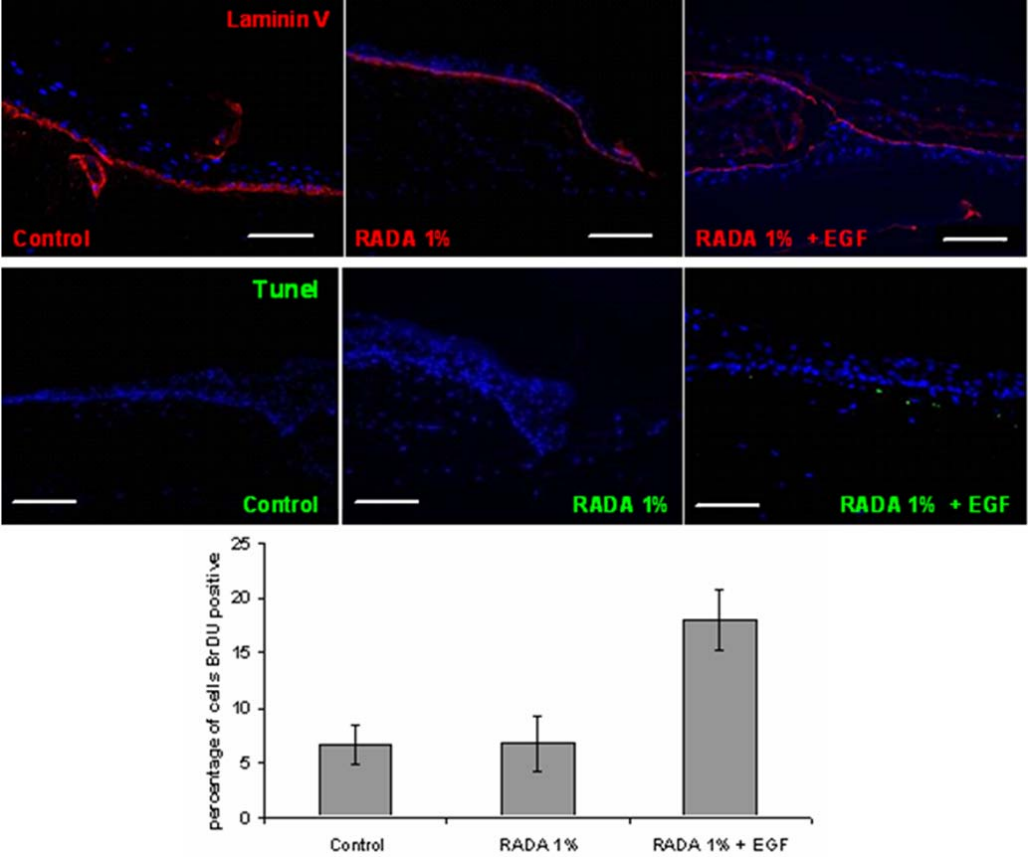
Control RADA16-I covered and RADA16-I-EGF-covered (10 µL/mL), specimens were immunostained for Laminin 5. The *in situ* TUNEL assay was performed on wounded tissues and apoptotic cells are seen (FITC-green). The scale bars are100 µm. Graphics demonstrate the percentage of BrdU-positive cells that were counted in 3 sections per sample.

To assess the phenotypic properties of tissues following wounding in the presence and absence of SAP-EGF, we studied the expression and distribution of the basement membrane protein Laminin 5, as well as proliferation and apoptosis of cells in the epithelial tongues. Figure 7 depicts the immunostaining of the tissues 48 hours after wounding when grown in the presence of SAP's containing EGF (10 µg/mL). Localization of keratinocyte-derived laminin 5 demonstrated a normal distribution beneath epithelial tonghes at the epithelial-connective tissue interface when wound healing was accelerated by bioactive nanoscaffolds, that was similar to control wounds. In situ TUNEL assay showed that a very small number (1.2±0.3%) of apoptotic basal cells were present in the wound epithelium that was covered with the SAP-EGF that were similar to control wounds control wounds (0.8±0.5%) (non significant differences as assessed by Student's t-test). In addition, there were no apoptotic cells in the part of the epithelial tongue in contact with the SAP, demonstrating that the SAP's are not cytotoxic and do not induce cell death when in contact with the tissues. To measure the proliferation of keratinocytes in the epithelial tongue and in the wound margin, wounded tissues were pulsed with BrdU 6 hours prior to termination of the experiment and numbers of BrdU-positive nuclei were counted in triplicate samples following IHC with a BrdU antibody. We determined that the percentage of BrDU-positive cells was 18±4% when wounds were treated with SAP+EGF, whereas only 7±3% of cells were proliferating in control tissues and 6±4% in those treated with RADA16-I. The presence of EGF thus leads to an increase in cell proliferation that may be linked to the greater degree of wound closure seen upon application of RADA-EGF to the wound.

## Discussion

Integrated efforts by tissue engineers and biologists have facilited the development of a new generation of biomaterials that combine suitable physico-chemical properties with biocompatibility, tissue integration and bioactivity. Among the new generation of materials, self-assembled peptides (SAP) are biologically-inspired material scaffolds that have been shown to meet these three criteria [17]. In the current study, we have advanced the use of SAPs by demonstrating the biological activity of peptides incorporated into the scaffold and by using the altered amino acid sequences containing the RAD motif: RADA16-I, RADARADARADARADARADA that are similar to the ubiquitous integrin receptor-binding site RGD [18], [19], [20] that have previously shown to elevate cytocompatibility and biointegration [21].

SAP scaffolds consist of alternating amino-acids that contain 50% charged residues [17], [22] and are characterized by their periodic repetition of alternating ionic hydrophilic and hydrophobic amino-acids that spontaneously form β-sheets that have distinct polar and non-polar surfaces. The gelation process can be accelerated by changing to a neutral pH or by adding physiological concentrations of salt solutions. Advantages of SAP nanofiber hydrogels include their biocompatibility [21], [22], their capacity to efficiently respond to external stimuli under physiological conditions and an ability to maintain a high water content (ie 99.5% w/v) that may allow for the diffusion of a wide range of molecules while preventing dehydration of adjacent tissues. We have significantly advanced the potential therapeutic application of these scaffolds by showing that these properties allow their adaptation to cutaneous wounds to serve as a bioactive wound dressing. Significantly, when combined with EGF, these SAPs have been shown to augment healing of cutaneous wounds in 3D human skin equivalents (HSE) that mimic their *in vivo* counterparts.

The future use of SAP-EGF for wound therapy is further advanced by previous studies demonstrating the lack of immunogenicity and inflammation of implanted SAP scaffolds in rat, rabbit, goat, and hamster models (data not published). Biodegradation products of SAP do not induce toxicity *in vivo*, as 14C carbon radiolabeled RADA16-I, has been shown to be completely eliminated after degradation in rabbit model (data not published). These studies have been shown that a number of mammalian cell types, including human dermal fibroblasts, human keratinocytes, rat hepatocytes, mouse and rat neuronal cells, adhere to and integrate into SAP peptides [23], [24], and support cell proliferation and differentiation *in vitro* [23], [24]. Linked to our findings that SAP-EGF accelerates wound reepithelialization, these previous studies suggest that wound coverage with SAPs may enable dermal responses as well, such as the early organization and remodeling of the provisional wound matrix. Thus, the biocompatibility and lack of cytotoxicity induced by SAPs, combined with our observations that SAP did not induce apoptosis of wound keratinocytes in contact with them support their future application as wound dressings during the early stages of repair.

Most studies on wound healing have been conducted using 2D cultures (for review see [25]) that use scratch-wounding of a cell monolayer to monitor cellular response to injury. While these studies have allowed elucidation of the cellular factors involved in cell migration and proliferation following “scratch woundings,” as well as in response to ECM proteins such as collagens, fibronectin, vitronectin and laminin [26], [27] and/or polypeptide growth factors [28], [29], [30], these simple cultures lack the complexity of the wound healing microenvironment *in vivo*. It is known that biologically-meaningful signaling pathways, mediated by linking of adhesion and growth, function optimally when cells are spatially organized in 3D tissues but are uncoupled and lost in 2D culture systems [31]. Therefore, it was essential in the present study to more fully simulate wound repair by testing the wound response to SAP in human tissues which display the architectural features seen in cutaneous tissues *in vivo* in order to further understand the function of SAP-EGF in reepithelialization of wounded epithelium. To accomplish this, we have adapted SAPs to study reepithelialization and wound response in a tissue that mimics the temporal and spatial events that occur following wounding of human skin [32], [33], [34]. By incorporating SAP into these 3D tissue models, we have demonstrated efficacy and safety findings that strongly support its use as a future therapeutic wound healing agent.

SAP nanoscaffolds have been previously loaded with different types of small molecules [14], [15] that demonstrated slow release from the scaffold. However, no experiments have reported the release or activity of larger (>850d) and electrically charged molecules such as growth factors after incorporation into SAPs. Since the role of these scaffolds has never been tested in wound repair, we decided to load SAP scaffolds with a growth factor and to test its role in enhancement of wound healing. We chose to test the response of wounds to EGF, as it has previously been shown to have an effect on wound closure by altering cell motility and proliferation [1], [35]. EGF has been shown to act directly at the wound site [9], [36] and similar responses have been observed when EGF is provided directly to the wound by the animal through saliva [37]. The fact that, in our experiments, EGF is topically delivered directly to the wound strongly supports the use of SAPs in this application as it mimics the biological release of this growth factor from endogenous sources in the wound environment. This effect is emphasized by our observation that EGF is released from the SAP gel only when the gel is in contact with the HSEs. This result may be explained by the destruction of the SAP gels by enzymes such as proteases that are secreted into the wound to remodel the ECM leading to the subsequent release of EGF. Such active proteases are secreted from the underlying tissues [1], [38] and from the epithelial tongue itself, as previously demonstrated in the HSE [7]. Our observations demonstrate that a threshold dose of EGF was necessary to induce a wound healing response, as the EGF effect was detected only in the presence of 10 µg/mL of EGF in the SAP gels and was similar to the controls when the amount of EGF loaded in the SAP gels was 1 µg/mL (data not shown).

It is now clear that the coordinated effects of multiple growth factors are needed to direct wound closure in order to avoid undesirable responses such as keloid formation and scarring [39]. By demonstrating the efficacy of scaffold-mediated delivery of a topically-acting growth factor, the incorporation of multiple, synergetic growth factors into SAPs may prove to be feasible for topical wound therapy. For example, since wound infection is often linked to chronicity of wound repair, it may be possible to utilize multi-functionalized SAPs that incorporate antimicrobial molecules that could both provide anti-bacterial factors, as well as wound healing stimulants such as EGF, to provide wound protection while stimulating wound closure. Such multi-functionalized bio-active nanomaterials will greatly advance the field of wound healing and require future testing in biologically-relevant, *in vitro* 3D tissue models and *in vivo*.

## Materials and Methods

### Preparation of Peptide Nanofibers

The peptide RADA16-I, [COCH3]-RADARADARADARADA-[CONH2] (molecular weight 1,712), was commercially synthesized and purified (Massachusetts Institute of Technology Biopolymers Laboratory). The solution of RADA16-I was prepared by dissolving the peptide powder with Milli-Q water. Final concentration of the peptide was 6 mM or 1% (10 mg/ml). The peptide solution was sonicated for 2 hours min with an ultrasonic cleaner (50T, VWR Scientific) before each measurement at the maximal power setting. The human recombinant EGF (Invitrogen-Gibco) was added after sonication at two different concentrations (1 µg/ml or 10 µg/ml) before the experiments. The peptide solution was sonicated for 2 hours before being put in contact with the wound to trigger gelation. EGF was added to the peptide solution after that sonication step to avoid any modification in the protein structure, well mixed by aspiring refouling. We added 100 µL of peptide solution on top of the wounded HSE.

### Cells

Human dermal fibroblasts, HDF, used for skin–equivalent cultures and scratch wound healing assay were derived from newborn foreskins using a combination collagenase and Trypsin/EDTA as previously described [40]. HDFs were grown in DMEM (Gibco, BRL) supplemented with 10% fetal bovine serum (Hyclone Laboratories), Penicillin—streptomycin (Sigma), and HEPES (Sigma). Cells were usually seeded at a density 5×10^4^ cells/ml (∼10% confluent) for extended cell passage and cultures were sequentially passaged when cell density reached confluence. Normal human keratinocytes (NHK) were isolated from newborn foreskin by the method of Rheinwald and Green [41] and maintained in keratinocyte medium described by Wu et al. [42]. Cultures were established through trypsinization of foreskin fragments and grown on irradiated 3T3 fibroblasts. 3T3 cells were maintained in DMEM (Gibco, BRL) containing 10% bovine calf serum (Hyclone Laboratories). All cells were grown at 37°C in 7.5% CO2.

### Fabrication of human skin-equivalent wound healing model

Skin-equivalent cultures were prepared and wounded *in vitro* as previously described [43]. Briefly, to construct the collagen matrix, HDF cells were added to neutralized Type I Collagen (Organogenesis, Canton, MA) to a final concentration of 2.5×10^4^ cells/ml; 3 ml of this mixture was added to each 35 mm well insert of a 6-well plate and incubated for 7 days in media containing DMEM and 10% fetal calf serum until the collagen gel showed no further shrinkage. At this time, of 5×10^5^ NHKs were plated directly on a raised, mesa-like area is seen in the center of the contracted collagen gel. Cultures were submerged in low calcium epidermal growth media for 2 days, submerged for 2 days in normal calcium epidermal growth media and raised to the air-liquid interface by feeding from below at 37°C in 7.5% CO_2_. Skin-equivalent cultures were wounded 7 days after keratinocytes were seeded onto the collagen matrix. One week before cultures were to be wounded, an additional collagen matrix was fabricated as described above. This was used as the substrate onto which the wounded skin-equivalents were transferred. To generate wounds, the skin-equivalent culture was removed from the insert membrane using 1.5 cm punch, and a 4 mm punch was used to incise the epidermis and collagen matrix. Wounded skin-equivalents were transferred onto the 7d-old collagen matrix. Wounded cultures were maintained at the air-liquid interface for 24h, and 48h at 37°C in 7.5% CO_2_ to monitor reepithelialization.

### Immunofluorescence

Specimen were frozen in embedding media (Triangle Biomedical, Durham, NC) in liquid nitrogen vapors after being placed in 2 M sucrose for 2 hr at 4_C. Tissues were serial-sectioned at 6 µm and mounted onto slides. Tissue sections were washed with PBS, blocked with 10 lg/ml goat IgG, 0.05% goat serum and 0.2% BSA vol/vol in PBS without fixation. Sections were incubated with monoclonal antibody to Involucrin (Abcam, Cambridge, MA) that was detected with Alexa 488-conjugated goat anti-rabbit IgG (Molecular Probes, Eugene, OR). Laminin V was detected using the GB-3 monoclonal antibody [44] directed against the intact heterotrimeric molecule (gift of Dr. G. Meneguzzi). Slides were coverslipped with Vectashield containing 1 lg/ml DAPI (Vector Laboratories) and fluorescence was visualized using a Nikon Eclipse microscope and double exposure photomicroscopy was performed using FITC and Texas Red filters.

### Proliferation and apoptosis assay

Prior to harvesting, skin-equivalents or collagen gels were labeled with a 6 h pulse of BrdU (Sigma, St. Louis, MO) at a final concentration of 10 µM. Tissues were harvested and frozen in embedding medium (Triangle Biomedical, Durham, NC) after being placed in 2 M sucrose overnight at 4°C. Frozen tissues were serially sectioned at 8 µm and stained with monoclonal antibodies against BrdU (Boehringer-Mannheim, Indianapolis, IN) and counterstained with DAPI (Vector, Burlingame, CA). For apoptosis detection, skin specimens were fixed in 10% neutral buffered formalin and in situ TUNEL assay (Roche diagnostics) was performed in paraffin sections. Fluorescence was visualized using a Nikon eclipse 80i microscope equipped with the EXFO Photonics X-CITE 120 Fluorescence Microscope Illumination System. Pictures were taken using Diagnostic Instruments SPOT RT Camera. The number of BrdU-positive cells was determined and expressed as a percent of total cells in the basal layer. Two independent experiments with a minimum of 3 observations for each condition were analyzed.

### Histology analysis

For routine light microscopy, skin-equivalent cultures were fixed in 4% neutral buffered formalin, embedded in paraffin, and serially sectioned at 8 µm. Sections were stained with hematoxylin and eosin, visualized and measured using a Nikon eclipse 80i microscope equipped with Diagnostic Instruments SPOT RT Camera.

### ELISA test assay

The RADA16-I solutions were prepared at 1%, EGF was then added (at 10 µg/mL) and mixed with the pipette. 100 µL of RADA16-I solution containing EGF was put in the bottom of an eppendorf tube, 900 µL PBS 1X were added on top of the peptide solution. At different times, 100 µL of PBS were taken and replaced by fresh PBS. For the in-vitro experiments, 100 µL of RADA16-I solution containing EGF were put directly on top of the wound. In this case 100 µL of medium surrounding the well with the wound, were taken at different times and replaces by fresh medium. The samples were then frozen and kept at −20°C until assayed for the cytokine levels using ELISA kits specific for Human EGF (Quantikine, R&D Systems, Minneapolis, MN). Serial dilutions were performed to determine cytokine concentrations by comparison with the standard according to the manufacturer's instruction.
